# Delayed functional maturation of natural regulatory T cells in the medulla of postnatal thymus: role of TSLP

**DOI:** 10.1186/1471-2172-7-6

**Published:** 2006-04-03

**Authors:** Qi Jiang, Hua Su, Geoffry Knudsen, Whitney Helms, Lishan Su

**Affiliations:** 1 Lineberger Comprehensive Cancer Center, School of Medicine, The University of North Carolina at Chapel Hill, Chapel Hill, NC 27599, USA; 2 Department of Microbiology and Immunology, School of Medicine, The University of North Carolina at Chapel Hill, Chapel Hill, NC 27599, USA; 3 Curriculum in Genetics and Molecular Biology, School of Medicine, The University of North Carolina at Chapel Hill, Chapel Hill, NC 27599, USA

## Abstract

**Background:**

Generation of functional CD4^+^CD8^-^CD25^+ ^regulatory T cells (Treg) in the murine thymus depends on FoxP3. Removal of the thymus from neonatal mice has been shown to result in a multiple organ autoimmune disease phenotype that can be prevented by introducing the FoxP3^+ ^Treg population to the animal. It has therefore, been proposed that functional FoxP3^+ ^Treg cells are not made in the neonatal thymus; however, it remains unclear when and where functional FoxP3^+^CD4^+^CD8^-^CD25^+ ^thymocytes are generated in postnatal thymus.

**Results:**

We report that neither FoxP3 mRNA nor protein is expressed in CD4^+^CD8^-^CD25^+^, or CD4^+^CD8^-^CD25^- ^thymocytes until 3–4 days post birth, despite the presence of mature CD4^+^CD8^-^CD25^+/- ^thymocytes in the thymus by 1–2 days after birth. FoxP3^-^CD4^+^CD8^-^CD25^+ ^thymocytes from day 2 newborn mice show no Treg activity. Interestingly, we are able to detect low numbers of FoxP3^+ ^thymocytes dispersed throughout the medullary region of the thymus as early as 3–4 days post birth. Expression of FoxP3 is induced in embryonic day 17 fetal thymus organ culture (FTOC) after 4–6 days of in vitro culture. Treatment of FTOCs with thymic stromal derived lymphopoietin (TSLP) enhanced expression of FoxP3, and blocking the TSLP receptor reduces FoxP3 expression in FTOC. Furthermore, TSLP stimulates FoxP3 expression in purified CD4^+^CD8^- ^thymocytes, but not in CD4^+^CD8^+^, CD4^-^CD8^+ ^and CD4^-^CD8^- ^thymocytes.

**Conclusion:**

Expression of FoxP3 or Treg maturation is ontogenically distinct and kinetically delayed from the generation of CD4^+^CD8^-^CD25^+ ^or CD4^+^CD8^-^CD25^- ^thymocytes in the postnatal thymus. TSLP produced from medullary thymic epithelia cells (mTEC) contributes to the expression of FoxP3 and the maturation of natural regulatory T cells. Overall, these results suggest that the development of Treg cells requires paracrine signaling during late stages of thymocyte maturation that is distinct from signaling during positive or negative selection.

## Background

It has been well-documented that the thymus plays a central role in deleting self-reactive T cells via negative selection. In addition, T cells with suppressive activity are generated in the thymus to regulate peripheral immunity [1,2]. Removal of the thymus from 3 day old neonatal mice leads to multiple organ autoimmune disease in some strains of mice [3-6]; however, removal of the thymus from mice 7 days old or older causes little or no autoimmunity. These observations have led to the proposal that T cells with suppressive activity in the thymus are either not generated in or fail to emigrate from the thymus in neonatal mice. Indeed, transfer of a subset of adult CD4^+^CD8^-^CD25^+ ^thymocytes (natural regulatory T or Treg cells) into day 3 thymectomized mice prevents autoimmune disease [7-9].

Natural CD4^+^CD25^+ ^regulatory T cells are present in the thymus and peripheral lymphoid organs [9,10]. Although studies have clearly shown that Treg cells are generated in the thymus, these cells can also be derived from mature T cells in the peripheral organs [11,12]. While the molecular mechanisms of Treg lineage development are poorly understood, recent genetic studies in both mice and humans have identified Scurfin or FoxP3, a forkhead family transcription factor, as a master determinant of Treg development and function [13-18]. Mice carrying mutations in the FoxP3 gene (scurfy mice) exhibit lymphoproliferative diseases and autoimmune phenotypes. These mice have been found to lack functional Treg cells. Similarly, human patients with mutations in the FoxP3 gene develop a multiple organ autoimmune disorder known as IPEX which is consistent with a lack of Treg cells. Finally, ectopic expression of FoxP3 in naïve CD4^+^CD25^- ^T cells causes them to convert to Treg cells [15,17], and targeted inactivation of FoxP3 in mice leads to loss of Treg development [16,18]. The preferential expression of FoxP3 in Treg cells and its deterministic activity in Treg lineage development make it the best marker of natural Treg cells, although the regulation of its expression and the mechanisms of its function in Treg are not yet elucidated.

According to the affinity/avidity model, CD4^+^CD8^+ ^DP thymocytes in the cortex or the cortical-medullary junctions (CMJ) that interact with self-MHC at low levels are rescued from death, thus positively selected. Alternatively, thymocytes are induced to die by negative selection if the interactions between thymocytes and thymic epithelial cells (TEC) or antigen presenting cells (APC) result in signaling above a certain threshold. These types of high-affinity interactions with self antigen are most efficiently mediated by APC, and usually occur at the CMJ. It has been proposed that induction of FoxP3 and maturation of the Treg lineage represent a separate lineage in the thymus due to selection signals that fall between those of positive and negative selection [19-22]. This hypothesis, however, has not been clearly verified. Interestingly, FoxP3^+^CD4^+^CD25^+ ^T cells have been induced in peripheral organs by prolonged exposure to their cognate antigens, suggesting that natural Treg-like cells can also be generated in a thymus-independent fashion [12,23].

We have investigated the kinetics of FoxP3 expression and generation of CD4^+^CD8^-^CD25^+ ^thymocytes in thymus organs of mice at various ages after birth. We demonstrate that neither FoxP3 mRNA nor protein is significantly expressed in CD4^+^CD8^-^CD25^+^, CD4^+^CD8^-^CD25^- ^or other thymocytes until 3–4 days post birth, despite the presence of CD4^+^CD8^-^CD25^+ ^thymocytes in the thymus of 1–2 day old neonatal mice. FoxP3 expression is induced at 3–4 days after birth and detected in both CD4^+^CD8^-^CD25^+ ^and CD4^+^CD8^-^CD25^- ^thymocytes. As expected, FoxP3^-^CD4^+^CD8^-^CD25^+ ^thymocytes from day 2 newborn mice proliferate in vitro and show no suppressive Treg activity. While an increasing percentage of CD4^+^CD8^-^CD25^+ ^thymocytes expresses FoxP3 from day 3 (21%) to day 8 (59%), a small population (<1%) of CD4^+^CD8^-^CD25^- ^thymocytes also expresses FoxP3. Almost all FoxP3^+ ^thymocytes are detected in the medullary region of the thymus even in 3–4 day old mice. Expression of FoxP3 is induced in embryonic day 17 fetal thymus organ culture (FTOC) 4–6 days after culture in vitro. Treatment of FTOC with thymic stromal derived lymphopoietin (TSLP) enhances expression of FoxP3. This expression correlates with increased maturation of FoxP3^+^CD4^+^CD25^+ ^thymocytes. Blocking the TSLP receptor significantly reduces FoxP3 expression in FTOC. TSLP stimulates FoxP3 expression in purified CD4^+^CD8^- ^thymocytes, but not in CD4^+^CD8^+^, CD4^-^CD8^+ ^or CD4^-^CD8^- ^thymocytes.

## Results

### Delayed expression of FoxP3 in CD4^+^CD25^+ ^thymocytes from thymus organs of neonatal mice

**Figure 1:**
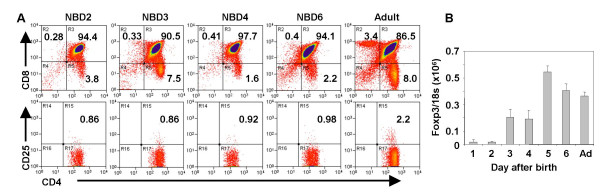
**Distinct development of SP4 (CD4^+^CD8^-^CD25^+/-^) cells and FoxP3 gene expression in new born thymus organs**. *A*: Comparable levels of CD4^+^CD8^-^CD25^+ ^thymocytes are present in day 1–8 neonatal and adult thymus. Neonatal thymus organs were analyzed for CD4, CD8 and CD25 by FACS. Relative percentages of CD4^+ ^thymocytes and CD4^+^CD8^-^CD25^+ ^thymocytes are indicated. Shown are neonatal mice from a representative litter of at least three independent experiments. *B*: FoxP3 mRNA is not expressed in neonatal thymocytes until 3 days after birth. Total cells from the newborn thymus were harvested at indicated times after birth and analyzed for relative expression of FoxP3 (FoxP3/18S) by real-time PCR. SD is derived from duplicate samples and shown as error bars. At least 4 litters of newborn mice were analyzed with similar results.

We investigated the ontogenic development of Treg cells in thymus organs from newborn mice. Comparable percentages of CD4^+^CD8^- ^thymocytes with Treg phenotypes (CD4^+^CD8^-^CD25^+^) are detected in day 1–8 neonatal thymus organs (Fig. 1A and Table 1). When measured by real-time PCR in thymocytes from neonatal mice, FoxP3 mRNA expression was not detected in thymocytes from 1 and 2 day old mice. Significant levels of FoxP3 expression were detected in thymocytes from the thymus of mice 3 days or older (Fig. 1B and data not shown). These data show that though CD4^+^CD25^+ ^thymocytes are present in 1–2 day old mice, FoxP3 is not expressed until 3 days after birth.

**Table 1:** FoxP3^+^CD4^+^CD25^+ ^thymocytes in neonatal mice ^a^

**# Cells (10^3^)**	**day 1**	**day2**	**day 3**	**day 4**	**day 5**	**day 6**	**day 8**	**Adult**
Total Thymocyte	1,005 ^b ^+/-177	3,390 +/-410	6,210 +/-127	9,410 +/-2,107	32,600 +/-18,668	31,000 +/-12,021	57,700 +/-2,263	75,900 +/-12,304
CD4^+^CD8^-^	13 +/-1.9	117 +/-36.4	457 +/-44.5	350 +/-16.2	828 +/-526	732 +/-361	1,738 +/-256	5,606 +/-1,751
CD4^+^CD25^+^CD8^-^	0.1 (.82%)^C ^+/-0.02	0.9 (.86%) +/-0.6	3.1 (.86%) +/-0.3	2.6 (.92%) +/-0.2	3.7 (.76%) +/-2.2	4.5 (.98%) +/-1.9	18.1 (.86%) +/-0.3	91.1 (2.2%) +/-27
Foxp3^+^CD4^+^CD25^+^CD8^-^	0	0	0.8 (21%)^d ^+/-0.1	0.9 (37%) +/-0.1	1.6 (43%) +/-0.9	1.9 (43%) +/-0.7	10.8 (59%) +/-0.1	64.5 (71%) +/-17.9

^a ^Total thymocytes from day 1–8 newborn or adult mice were stained with CD4, CD8, CD25 and anti-FoxP3 antibody and analyzed by FACS. Data is a summary of three independent analyses. ^b^: Average cell number +/- standard deviations (SD). ^c^: % CD25^+ ^of CD4^+^CD8^- ^thymocytes. ^d^: % FoxP3^+ ^of CD4^+^CD25^+^CD8^- ^thymocytes.

**Figure 2:**
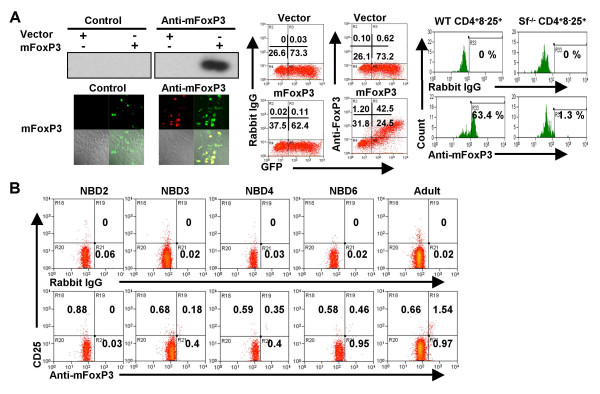
**Delayed FoxP3 expression in CD4^+^CD8^-^CD25^+/- ^cells in neonatal thymus**. *A*: Generation and characterization of the anti-FoxP3 antibody.293T cells were transfected with either vector or pHSPG-mFoxp3. FoxP3 was specifically detected by Western blot with the anti-FoxP3 antibody (top left), immunofluorescence (IF) staining (lower left), by FACS analysis (middle). The anti-FoxP3 antibody specifically stained intracellular FoxP3 in CD4^+^CD8^-^CD25^+ ^Treg cells from wild type, but not scurfy, mice (right). *B*: Expression of FoxP3 protein is not detected in CD4^+^CD8^-^CD25^+ ^thymocytes until 3 days after birth. Percentages of CD4^+^CD8^-^CD25^+ ^and CD4^+^CD8^-^CD25^- ^thymocytes (gate on the CD4^+^CD8^- ^population from Fig. 1A) that expressed FoxP3 are shown, with the control IgG as background.

To analyze if and when the FoxP3 protein is expressed in new born thymocytes, we generated an anti-FoxP3 antibody. Using 293T cells transfected with mouse FoxP3, we showed that this antibody could be used to detect FoxP3 by Western, immunofluorescence staining and FACS analysis (Fig. 2A). Anti-FoxP3 antibodies specifically stained intracellular FoxP3 in CD4^+^CD25^+ ^Treg cells isolated from wild type, but not scurfy, mice by FACS (Fig. 2A, right). Using the anti-FoxP3 antibody, we showed that the FoxP3 protein was not detected in the CD4^+^CD8^-^CD25^+ ^thymocytes from 1 and 2 day old neonatal mice as determined by FACS (Fig. 2B). Significant levels of FoxP3 were first detected in CD4^+^CD25^+ ^and CD4^+^CD25^- ^cells from the 3 day old thymus (21% FoxP3^+ ^cells in CD4^+^CD8^-^CD25^+ ^thymocytes), reached high levels at 6–8 days (41 % in day 6 and 59 % in day 8), and were further elevated and maintained in the adult thymus (70 % FoxP3^+ ^cells in CD4^+^CD8^-^CD25^+ ^thymocytes) (Fig. 2B and Table 1). Consistent with recent reports in the adult thymus [24,25], a small population (<1%) of CD4^+^CD8^-^CD25^- ^thymocytes also expressed FoxP3 (Fig. 2B, 4C and data not shown). Therefore, expression of FoxP3 seems to lag behind the generation of CD4^+^CD8^-^CD25^+ ^thymocytes, indicating that maturation of FoxP3^+ ^Treg thymocytes are delayed relative to mature single positive thymocytes (CD4^+^CD8^-^CD25^+ ^or CD4^+^CD8^-^CD25^-^). This delay in maturation and FoxP3 expression in the newborn thymus may help to explain the preferential lack of Treg cells in day 3 thymectomized mice.

### CD4^+^CD8^-^CD25^+ ^thymocytes from day 2 newborn mice are not functional Treg cells

**Figure 3:**
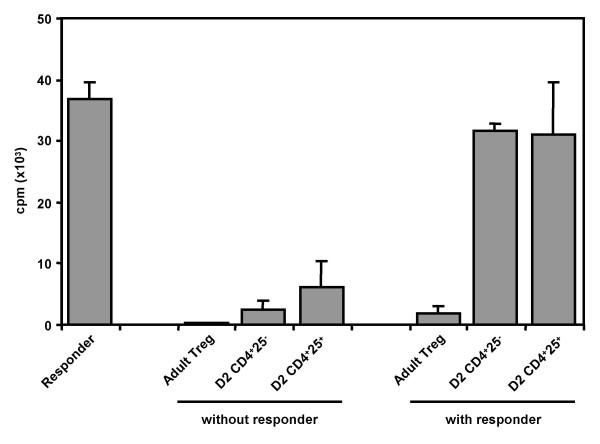
**CD4^+ ^CD8^- ^25^+ ^thymocytes from day 2 thymus are not functional Treg cells**. CD4^+^CD8^-^CD25^+ ^thymocytes from day 2 newborn thymus or mature Treg cells from adult mice were purified and activated alone or mixed at 1:2 ratio with fixed number of CD4^+^CD25^- ^responder T cells. Proliferation was determined after 3 days of culture using ^3^H-thymidine incorporation. Error bars indicate SD of triplicate samples.

To investigate if the CD4^+^CD8^-^CD25^+ ^thymocytes in day 2 newborn mice are functional Treg cells, we sorted this population and analyzed their proliferation and suppressive capacity in vitro. As shown in Fig. 3, adult Treg cells expressing FoxP3 showed no proliferation and efficiently suppressed responder T cells. In contrast, CD4^+^CD8^-^CD25^+ ^thymocytes from day 2 newborn mice proliferated in response to TCR stimulation in vitro and did not suppress proliferation of CD4^+^CD25^- ^responder T cells, indicating that they were not functional Treg cells.

**Figure 4:**
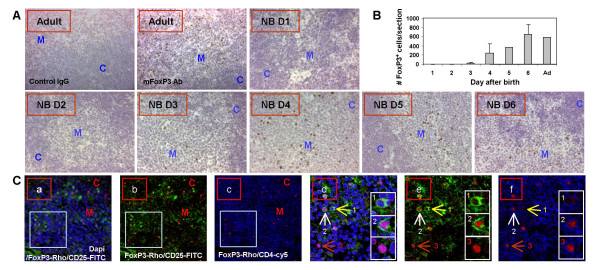
**Kinetics and localization of FoxP3^+ ^cells in neonatal thymus organs**. *A*: FoxP3 expression in 1–6 day old newborn or adult thymus lobes was analyzed by immunohistochemical staining with an anti-FoxP3 antibody. Rabbit IgG was used as control. No FoxP3^+ ^cells could be detected in the 1–2 day old newborn thymus. In the 3–6 day old thymus, as in the adult thymus, the majority of FoxP3^+ ^cells were found in the medullary region. NB: newborn, C: Cortex, M: Medulla. *B*: Total FoxP3^+ ^cell numbers from each section were determined by counting 3 different medulla regions/sections and summarized. *C*: Immunofluorescence phenotyping of FoxP3^+ ^cells in the thymus. a and d: Dapi (Blue), FoxP3 (Red) and CD25 (Green). b and e: FoxP3 (Red) and CD25 (Green). c and f: FoxP3 (Red) and CD4 (Blue). C: cortex; M: medulla. In d-f: 1 indicates a CD4^+^CD25^+^FoxP3^- ^cell; 2, a CD4^+^CD25^+^FoxP3^+ ^and 3, a CD4^+^CD25^-^FoxP3^+ ^cell.

### Localization of FoxP3^+ ^Treg cells in the thymus of neonatal and adult mice

To determine the localization and phenotype of FoxP3^+ ^Treg cells in the neonatal thymus, we performed immunohistochemical and immunofluorescence staining. As shown in Figure 4A, FoxP3^+ ^cells were not detected in the thymus of 1–2 day old neonatal mice. Low numbers of FoxP3^+ ^cells were first detectable in the thymus of 3 day old mice. The number of FoxP3^+ ^cells increased significantly in the 4–5 day old thymus and reached adult levels in the 6 day old thymus (Fig. 4A and 4B). Interestingly, almost all FoxP3^+ ^cells are sparsely localized in the medullar region of the thymus (Fig. 4A and 4C). This observation holds true even in 3–4 day old mice, suggesting the importance of the thymic medulla in providing the paracrine factors to induce FoxP3 expression and Treg maturation.

### Enhanced expression of FoxP3 by TSLP in fetal thymus organ culture

**Figure 5:**
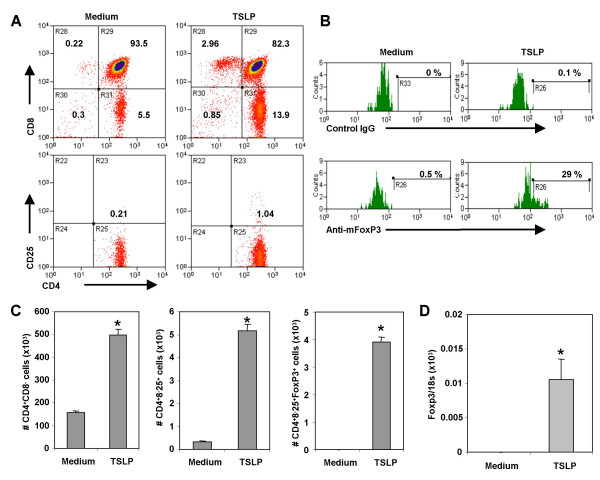
**TSLP enhances FoxP3 induction in FTOC**. *A*: Induction of FoxP3 expression in FTOC with E17 fetal thymus. Total thymocytes were harvested at 2, 4 and 6 days after E17-FTOC and analyzed for relative expression of FoxP3 (FoxP3/18S) by real-time PCR. SD is derived from duplicate samples and shown as error bars. At least 3 independent experiments were performed. *B*: Enhancement of FoxP3 expression by exogenous TSLP in FTOC. FTOCs treated with TSLP (100 ng/ml) were harvested after 4 days and analyzed as above. Shown are representative data from at least three independent experiments. *C*: Endogenous TSLPR activity is required for induction of FoxP3 expression. FTOCs were treated with control IgG or anti-TSLPR antibody. FoxP3 expression after 4 days in culture was analyzed by real-time PCR as above. Error bars indicate standard errors from duplicate samples. * indicates p < 0.05. *D*: FACS analysis of FoxP3 expression in thymocytes from FTOC stimulated with exogenous TSLP. FTOC treated with TSLP was harvested at 4 days post FTOC as above. CD4^+^CD8^- ^thymocytes were gated for analysis of CD4 vs. CD25 and CD25 vs. FoxP3 or isotype control. Shown are representative data from three independent experiments.

Thymic stromal derived lymphopoietin (TSLP) is a type I cytokine predominantly produced by thymic medullary epithelial cells and has been recently shown to modulate human DC and T cell responses [26-28], and to promote proliferation of CD4^+ ^T cells in mice [29]. Because of this finding and its expression in the medulla, we decided to test the hypothesis that TSLP is also involved in the induction of Treg development in mouse thymus organs. We investigated the kinetics of FoxP3 expression in thymus organs from day 17 embryos cultured in vitro (FTOC). Expression of FoxP3 was not detectable in day 0 or day 2 FTOC, but was detected at significant levels after 4–6 days in culture (Fig. 5A). We therefore used the E17 FTOC model to investigate paracrine signals that contribute to the expression of FoxP3 in the thymus. When tested in the FTOC model, TSLP enhanced the expression of FoxP3 in the thymus (Fig. 5B). While the total number of thymocytes was not significantly changed, TSLP treatment did significantly enhance maturation of CD4^+ ^and CD8^+ ^single positive thymocytes. In addition, both the relative percentage and total number of CD4^+^CD25^+ ^thymocytes were enhanced (Fig. 5D). These observations indicate that exogenous TSLP promotes maturation of thymocytes, including the CD4^+^CD8^-^CD25^+^FoxP3^+ ^natural Treg cells in the thymus.

To demonstrate that endogenous TSLP-mediated signaling is required in the thymus for FoxP3 expression, we used an antibody that blocks the TSLP receptor (TSLPR) in the FTOC model. Blocking the TSLPR with this antibody inhibited FoxP3 expression, whereas a control antibody showed no effect (Fig. 5C and data not shown). This experiment suggests that TSLP-mediated signaling is involved in the expression of FoxP3 and maturation of Treg thymocytes in the thymus. Similar fractions of FoxP3^+^CD4^+^CD25^+ ^thymocytes were, however, found in TSLPR-null and age-matched wild type mice (data not shown). TSLP, therefore, contributes to, but is not absolutely required for, the maturation of FoxP3^+^CD4^+^CD25^+ ^Treg cells in the murine thymus.

### TSLP enhances FoxP3 expression in purified CD4^+^CD8^- ^thymocytes in vitro

**Figure 6:**
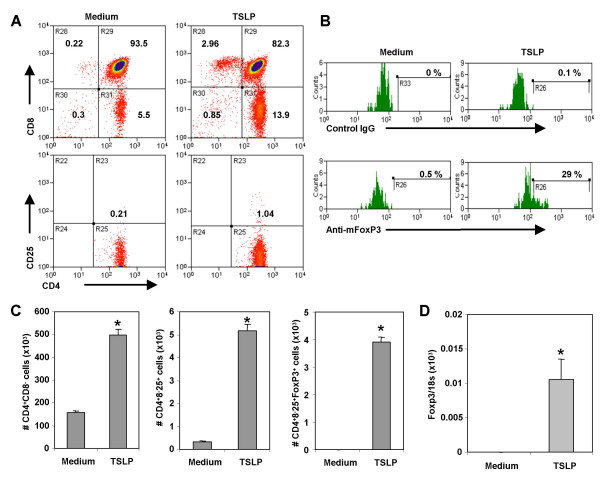
**TSLP interacts with thymocytes to affect FoxP3 expression and Treg maturation**. Thymocytes from day 2 newborn mice were cultured with or without TSLP in mFTOC medium for 2 days. Cells were harvested and analyzed by FACS and real-time PCR. *A and B*: TSLP increased maturation of SP, CD4^+^CD25^+ ^thymocytes and FoxP3 expression. Shown are data repeated in at least three independent experiments. *C*: Absolute numbers of CD4^+^CD8^-^, CD4^+^CD8^-^CD25^+ ^and FoxP3^+^CD4^+^CD8^-^CD25^+ ^in cultures started with initial 10 × 10^6 ^total thymocytes from day 2 neonatal mice. Data show mean and standard deviation from three different experiments. * indicates p < 0.05 by student's t test. *D*: TSLP induced FoxP3 expression in thymocytes from day 2 neonatal mice. Thymocytes were cultured with or without TSLP for 2 days and analyzed by real-time PCR as above. * indicates p < 0.05.

To investigate whether TSLP interacts directly with thymocytes to induce FoxP3 expression or indirectly via interactions with other cell types such as DCs in the thymus as reported in humans [26-28], we isolated thymocytes from 2 day old mice and treated them with TSLP in vitro for 2 days. As seen above in FTOC, TSLP promoted the maturation of thymocytes including the CD4^+^CD8^-^CD25^+^FoxP3^+ ^natural Treg thymocytes (Fig. 6A, 6B and 6C). In the absence of TSLP, freshly isolated thymocytes from 2 days old thymus did not express detectable levels of FoxP3 after 2 days in culture. TSLP induced FoxP3 expression at both protein and mRNA levels in cultured thymocytes (Fig. 6B and 6D). Using TSLP receptor mutant thymocytes, we couldn't detect any enhancement of FoxP3 expression by TSLP, proving that TSLP was indeed working through the TSLP receptor in this system (Fig. 8). The data suggest that, in contrast to human TSLP which mediates DC maturation and indirectly induces Treg activity in the human thymus, mouse TSLP can interact directly with mouse thymocytes to promote maturation of FoxP3^+^CD4^+^CD25^+ ^thymocytes and to enhance FoxP3 gene expression.

**Figure 7:**
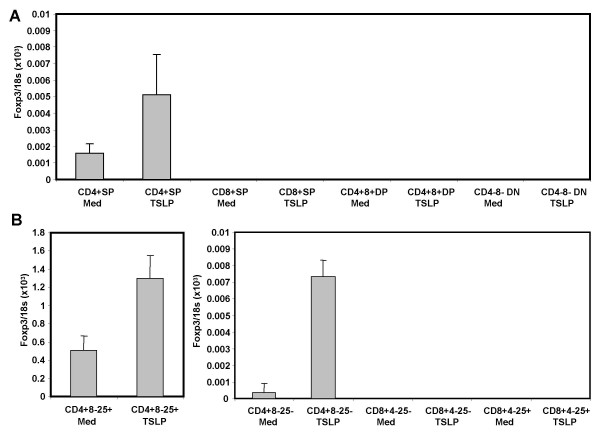
**TSLP specifically enhances FoxP3 expression in CD4^+^CD8^- ^thymocytes**. *A*: TSLP induced expression of FoxP3 in SP4, but not DP, DN and SP8 thymocytes. Purified adult thymocyte subsets were cultured with TSLP and analyzed for FoxP3 expression by RT-PCR. Relative expression of FoxP3 is shown.*B*: TSLP induced expression of FoxP3 in both CD4^+^CD8^-^CD25^- ^and CD4^+^CD8^-^CD25^+ ^SP4 thymocytes. Purified thymocytes were cultured and analyzed as above. No FoxP3 gene expression is detectable with or without TSLP in CD4^-^CD8^+^CD25^+ ^or CD4^-^CD8^+^CD25^- ^SP8 thymocytes.

To test which thymocyte subpopulations directly respond to TSLP, we purified CD4^+^CD8^+ ^(DP), CD4^-^CD8^- ^(DN), CD4^+^CD8^- ^(SP4) or CD4^-^CD8^+ ^(SP8) thymocytes and treated them with TSLP in vitro for two days. TSLP enhanced expression of FoxP3 in SP4 thymocytes, but not in DP, DN or SP8 thymocytes (Fig. 7A). We further subdivided the SP4 and SP8 thymocyte populations based on their expression of CD25. TSLP enhanced FoxP3 expression by 10-fold in CD4^+^CD25^- ^thymocytes, and about 2-fold in CD4^+^CD25^+ ^thymocytes (Fig. 7B). In contrast, neither CD25^+ ^nor CD25^- ^SP8 thymocytes responded to TSLP. We also investigated if TSLP affects the proliferation or survival of thymocytes during the two day culture in vitro. Our results showed that TSLP treatment affected neither the proliferation nor the survival of CD4+CD25- or CD4^+^CD25^+ ^thymocytes (Fig. 9A and 9B). Together our data suggest that interaction of TSLP with its receptor can promote FoxP3 expression and CD4^+^CD25^+^FoxP3^+ ^Treg development in the thymus, at least partly via direct interaction with CD4^+ ^SP thymocytes.

**Figure 8:**
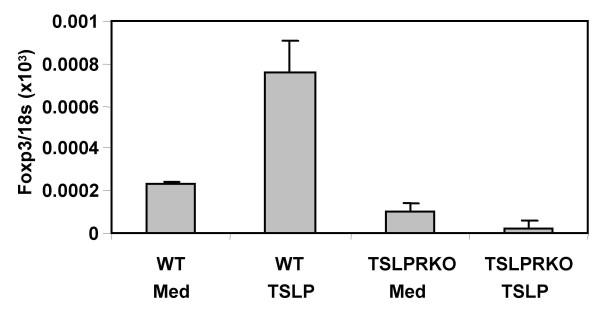
**TSLP receptor (TSLPR) is required for the enhancement of FoxP3 expression by TSLP**. Thymocytes from wild type or TSLPR-null mice (4–6 weeks of age) were cultured with or without TSLP (100 ng/ml) for 2 days and analyzed for relative expression of FoxP3 (FoxP3/18S) by real-time PCR. Error bars indicate SD derived from duplicate samples. Shown are data repeated in at least three independent experiments.

**Figure 9:**
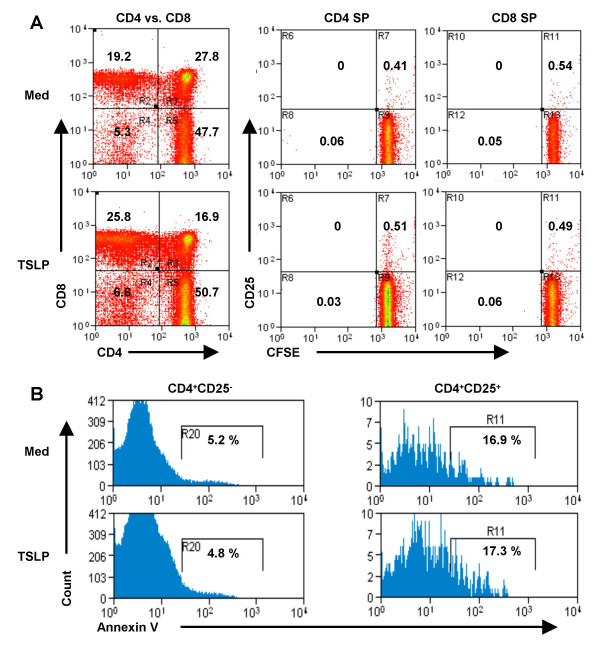
**TSLP does not affect the proliferation and survival of CD4^+^CD25^- ^or CD4^+^CD25^+ ^thymocytes in vitro**. A. Lack of proliferation of thymocytes in the absence or presence of TSLP. Total thymocytes from 4–6 weeks old mice were pre-labeled with CSFE, and then cultured with or without TSLP (100 ng/ml) for two days. Thymocytes were analyzed for CD4, CD8, CD25 and CFSE by FACS. Relative CFSE levels on CD25^- ^and CD25^+ ^SP thymocytes (CD4^+ ^SP and CD8^+ ^SP) were measured. No significant proliferation was detected in these thymocytes under the culture conditions. B. TSLP does not affect apoptosis of thymocytes. Total adult thymocytes were cultured with or without TSLP for 2 days. Annexin V^+ ^cells in the CD4^+^CD25^- ^and CD4^+^CD25^+ ^populations are shown by FACS analysis. Similar results were obtained in three independent experiments.

## Discussion

We report here that, although mature single positive thymocytes including CD4^+^CD25^+ ^thymocytes can be detected in day 1–2 neonatal mice, the FoxP3 gene is not expressed in the thymus until 3–4 days after birth and the expression dose not approach adult levels until after 6–8 days. FoxP3 expression was detected in both CD4^+^CD8^-^CD25^+ ^and CD4^+^CD8^-^CD25^- ^thymocytes. As expected, FoxP3^-^CD4^+^CD8^-^CD25^+ ^thymocytes from day 2 newborn mice show no Treg activity. Almost all FoxP3^+ ^thymocytes are detected in the medullary region of the thymus. In addition, we have demonstrated that TSLP enhances maturation of CD4^+^CD8^-^CD25^+ ^thymocytes as well as FoxP3 expression, and that blocking endogenous TSLPR in the thymus inhibits FoxP3 expression. Furthermore, TSLP interacts directly with CD4^+ ^SP thymocytes to affect FoxP3 expression and Treg development.

Our findings agree with a recent report showing that lower levels of FoxP3 mRNA were detected in CD4^+^CD25^+ ^T cells from 3 day old newborn mice [30]. This report, however, failed to clarify whether FoxP3 is expressed in 1–2 day old mice, and if all Treg cells from 3 day old mice express lower levels of FoxP3 mRNA or if a smaller fraction of these cells expresses the same level of FoxP3 mRNA as CD4^+^CD25^+ ^Treg cells from adult mice. We have demonstrated here that FoxP3 expression is not detectable in Treg-like cells from 1–2 day old mice, and that a smaller fraction of day 3 neonatal CD4^+^CD25^+ ^thymocytes expresses the FoxP3 protein at levels comparable to mature adult Treg cells.

As detected by immuno-staining of thymus tissues, both CD4^+^CD25^+^FoxP3^+ ^and CD4^+^CD25^-^FoxP3^+ ^cells were detected in the thymus (Fig. 3). FoxP3^+ ^thymocytes are induced in the 3–4 day old thymus, with a medullary distribution similar to that seen in the adult thymus. This is consistent with a recent report using the FoxP3-GFP knock-in mouse. In this model, expression of FoxP3 is detected by GFP [24,31]. Similar to our finding using anti-FoxP3 staining, the FoxP3-GFP^+ ^thymocytes start to be detectable in the medulla of the 3–4 day old thymus. These results suggest that CD4^+^CD25^+ ^thymocytes need paracrine signals provided by cells in the medullary region to become fully mature FoxP3^+ ^Treg thymocytes. As positive selection and negative selection of thymocytes occurs predominantly in the cortex and the CMJ, we would expect a preferential localization of emerging FoxP3^+ ^thymocytes in the cortex/CMJ if commitment to natural Treg and FoxP3 induction is closely related to the TCR-mediated positive/negative selection. The dispersed medullary distribution of FoxP3^+ ^thymocytes in 3–4 day old thymus organs supports the idea that induction of FoxP3 expression may be a post-selection event, involving unique cell types and/or cytokines in the medulla.

CD28-mediated signaling is required for the induction of FoxP3 and natural Treg generation [32]. The CMJ is enriched with APC that express the B7 ligands which signal to CD28. Other factors must, therefore, be provided in the medulla to lead to functional maturation of FoxP3^+ ^Treg thymocytes. It was recently reported that the Hassall's corpuscles in the human thymic medulla preferentially express the cytokine TSLP, which promotes maturation of DC and induces natural Treg generation [33]. The murine thymus does not have Hassall's corpuscle-like structures although mouse TSLP is also predominantly expressed by medullary TECs in the thymus [33]. We report here that exogenous mouse TSLP can enhance expression of FoxP3 and maturation of natural Treg cells in mouse FTOC, and that blocking TSLPR leads to reduced expression of FoxP3. We have detected TSLP gene expression by RT-PCR in 1–6 days old neonatal and adult thymus organs, but its expression kinetics is not correlated with functional FoxP3^+ ^Treg development (data not shown). Additional paracrine factors are probably involved.

**Figure 10:**
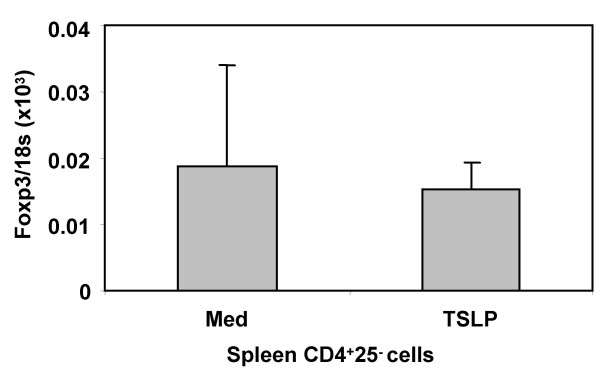
**TSLP does not induce Foxp3 expression in CD4^+^25^- ^spleen T cells**. CD4^+^25^- ^T cells purified from the spleen were cultured with or without TSLP (100 ng/ml) for 2 days and analyzed for relative expression of FoxP3 (FoxP3/18S) by real-time PCR. Error bars indicate SD derived from duplicate samples. Shown are data repeated in at least three independent experiments.

How does TSLP modulate maturation of CD4^+^CD25^+^FoxP3^+ ^Treg cells in the thymus? Although TSLP is expressed predominantly by thymic medullary stromal cells, its effect on T cells in mice has not been clearly established [34-36]. In transgenic mice over-expressing TSLP, development of B cells and T cells appears to be impaired, but myeloid cell numbers are increased [37]. In human studies, TSLP has been shown to modulate DC maturation to indirectly regulate T cell responses [26-28]. In contrast, mouse TSLP has been implicated in directly promoting TCR-mediated proliferation of mouse CD4^+ ^T lymphocytes both from the thymus and spleen [29]. Our study with purified CD4^+ ^thymocytes after two days in culture, in the absence of TCR stimulation, suggests that TSLP induces FoxP3 in CD4^+^CD8^- ^(both CD25^+ ^and CD25^-^) thymocytes, but not in CD4^+^CD8^+ ^DP or CD4^-^CD8^+ ^SP8 thymocytes. In addition, TSLP failed to induce FoxP3 expression in CD4^+^CD25^- ^T cells from the spleen under similar culture conditions (Fig. 10). We also showed that TSLP treatment affected neither the proliferation nor the survival of CD4^+^CD25^- ^or CD4^+^CD25^+ ^thymocytes during the two day culture in vitro (Fig. 9). Therefore, TSLP may have a unique effect on CD4^+ ^thymocytes to induce FoxP3 gene expression that is distinct from its activity in promoting CD4^+ ^T cell proliferation.

The receptor for TSLP consists of a heterodimer of the interleukin 7 receptor alpha (IL-7Rα) chain and the TSLP receptor (TSLPR) that resembles the cytokine receptor common gamma chain [35,38,39]. As suggested in previous reports with TSLPR mutant mice [29,36], TSLPR and other receptors/ligands functionally interact to modulate B cell development and T cell proliferation. TSLP-mediated signaling is unique among members of the cytokine receptor family in that activation of the transcription factor Stat5 occurs without detectable Janus kinase activation [40,41]. Interestingly, murine TSLPR mediates signals that are distinct from the Jak3 mediated signals seen with the common gamma chain [36]. This potentially unique cytokine signaling activity of TSLPR may be involved in modulating FoxP3 expression and CD4^+^CD25^+ ^Treg maturation in thymocytes. TSLP also appears to have different effects on pro-B cells of fetal and adult origin [42], suggesting distinct signaling networks in developing vs. mature cell types may determine their responsiveness to TSLP. A similar distinction may exist in the thymocytes versus peripheral mature T cells as, unlike what we have seen in the thymus, treatment of CD4^+^CD25^- ^T cells from the spleen with TSLP showed no effect on FoxP3 gene expression (Fig. 10).

Blocking TSLPR in FTOC only partially reduces the number of FoxP3^+^CD4^+^CD25^+ ^thymocytes. In addition, the frequency and function of FoxP3^+^CD4^+^CD25^+ ^Tregs in the spleen and lymph nodes of TSLPR-null adult mice are not significantly altered (Q. Jiang, G. Knudsen and L. Su, unpublished results). Thus, TSLP is involved in, but not absolutely required, for the process that regulates FoxP3 expression and Treg maturation. Other factors may compensate for the loss of TSLP signaling during embryogenesis or during Treg maturation. Paracrine factors known to be involved in Treg generation and function, including interleukin-10 and TGFβ, are also expressed in the thymus and may contribute to this process. For example, TGFβ has been reported to induce Treg and FoxP3 expression in mature CD4^+^CD25^- ^T cells [11,43]. Our preliminary results, however, indicate that TGFβ and IL-10 had very low or no effect on FoxP3 expression in the FTOC model (Q. Jiang and L. Su, unpublished results). It was recently reported that the cytokine common gamma chain γc is required for the expression of FoxP3 and Treg maturation [44]. In this context, the TSLPR and γc receptor may mediate similar down-stream signals such as STAT5 to activate FoxP3 gene expression and Treg maturation. It will be of interest to use STAT5a/b double KO mice [45,46] to test weather STAT5 is required for the expression of FoxP3 and Treg generation.

Regulatory CD4^+^CD25^+ ^T cells have been implicated in a number of pathologic processes including elevated levels of Treg cells in certain types of cancer [47-49] and infectious diseases [17,50-52], and reduced Treg levels in autoimmune diseases [9,53-56]. Novel therapies based on modulating Treg activity have great potential in treatment of these diseases and in promoting engraftment of allogeneic organ transplant. Elucidating how FoxP3 expression is modulated during the development of natural Treg cells in the thymus will be of great importance both for understanding the ontogenic mechanism of their development, and for shedding light on how to modulate their generation and expansion for use in therapeutic applications.

## Methods

### Mice, FoxP3 antiserum production and FACS analysis

B6/Ly5.1 and B6/Ly5.2 mice were purchased from the Jackson Laboratory and TSLP-R-deficient mice were provided by Dr. J. Ihle in St. Jude Children's Research Hospital in Tennessee. Scurfy mice (mutation of FoxP3 (*FoxP3*^*sf*^) genes) were kindly provided by Dr. VL. Godfrey at the University of North Carolina at Chapel Hill. All mice were housed at the University of North Carolina at Chapel Hill as described [57]. The peptide from the N-terminus of murine FoxP3 (C-LLGTRGSGGPFQGRDLRSGAH) was chosen to immunize rabbits for production of antibodies. Affinity purification of the antibodies was done with a SulfoLink Kit (Pierce Biotechology, Rockford, IL). Monoclonal antibodies to CD3, CD4, CD8α and CD25 (Caltag Laboratories, Burlingame, CA), Annexin V-APC (BD Biosciences, Mountain View, CA) were used for staining and the affinity purified FoxP3 antibody for intracellular staining. For proliferation analysis, total thymocytes from adult mice were labeled with CFSE (1.25 μM) in HBSS/5 % FBS, cells were washed twice, then cultured with or without TSLP (100 ng/ml) for 2 days before FACS analysis. Cells were analyzed on a FACSCalibur™ flow cytometer (BD Biosciences) as descried [58].

### Transfection

The PG-mFoxp3 plasmid was constructed by PCR of mFoxp3 into the pHSPG vector [57]. 293T cells were transfected with either empty vector or PG-mFoxp3 using Effectene Transfection Reagent (Qiagen, Valencia, CA).

### Western blot

293T cells transfected with either empty vector or pHSPG-mFoxp3 were lysed and resolved on a 10% SDS-PAGE. Gels were transferred to PVDF membranes (Amersham Pharmacia, NJ), and probed with either control, followed with the anti-rabbit-IgG-HRP and visualized using an ECL Kit (Amersham Pharmacia).

### Real-time PCR

RNA and cDNA were prepared with the cells to cDNA kit (Ambion, Inc, Austin, Texas). mRNA levels of FoxP3 and 18S were measured by TaqMan real-time PCR using the primers and ABI 7000 Sequence Detection System (Applied Biosystems Inc.). FoxP3 mRNA levels were normalized relative to 18S. All samples were run in duplicates and repeated in at least three independent experiments.

### Immunostaining

For immunohistochemical staining, paraffin-embedded sections of thymus organs from new born or adult mice were stained with the anti-FoxP3 antibody, goat anti-rabbit biotin and the Avidin-Biotin Complex (ABC) (Vector, CA) and substrate DAB (Pierce, IL). For immunoflurescence staining of 293T cells, after transfection with an vector expressing both FoxP3 and GFP on coverslips, cells were stained with pre-immune serum or anti-Foxp3 serum, followed by Rhodamine (TRITC)-conjugated goat anti-rabbit IgG (H+L) (Jackson ImmunoReasearch Laboratories, INC PA). For immunoflurescence staining of thymus organs, frozen sections were incubated with the anti-FoxP3 antibody followed by goat anti-rabbit IgG-Alexa Fluor 546 (Molecular Probes, CA), mouse CD25-Alexa Fluor 488 and mouse CD4-Alexa Fluor 647 (Caltag, CA). Nuclei were counterstained with DAPI. The sections were examined with a LeicaSP2 confocal microscope (Leica, Bannockburn, IL).

### E17 FTOC assays

Thymic lobes from day 17 embryos were cultured on the membranes at an interface between 5 % CO_2_-humidified air and mFTOC media (RPMI 1640 (BRL), 10 % fetal bovine serum, 50 μM 2-mercaptoethanol, 2 mM L-glutamine, 1 % nonessential amino acid (Life Technologies, Gaithersburg, MD), 10 mM HEPES, 1 mM sodium pyruvate solution (GIBCO BRL), 100 U/ml Penicillin and 100 mg/ml Streptomycin). In E17 thymus organ culture system, lobes were cultured with or without recombinant mouse TSLP (R&D Systems, Minneapolis, MN) or monoclonal rat anti-mouse TSLPR antibody (R&D Systems). Cells from thymic lobes were harvested at different days and analyzed by flow cytometer or real-time PCR.

### Newborn, adult thymocytes or adult splenocytes culture in vitro

Thymocytes from day 2 newborn mice or 2–3 month old adult mice were harvested. Different subpopulations of adult thymocytes and splenocytes were sorted on a Cytomation MoFlo FACS. Cells were cultured in FTOC media with or without recombinant mouse TSLP (R&D Systems). Two days later, cells were isolated and analyzed by PCR or on a FACSCalibur™ flow cytometer.

### Treg proliferation and suppression assay

Thymus organs from day 2 newborn or adult mice and spleens from adult mice were harvested. To isolate Treg cells from thymocytes, cells were stained with anti-CD4, anti-CD8 and anti-CD25 antibodies, CD4^+^CD8^-^CD25^+ ^and CD4^+^CD8^-^CD25^- ^cells were sorted on a Cytomation MoFlo machine. CD90^- ^APCs were purified on the autoMACS system using CD90 microbeads (Miltenyi Biotec, Auburn). The proliferation and suppression assay of Treg cells was performed as described [59]. Purified day 2 newborn CD4^+^CD8^-^CD25^+^, CD4^+^CD8^-^CD25^- ^cells (7.5 × 10^3^), or adult CD4^+^CD25^+ ^(7.5 × 10^3^) Treg are cocultured with CD4^+^25^- ^cells (1.5 × 10^4^) and irradiated (2100 rad) CD90-depleted splenocytes (1.5 × 10^4^) as APCs in the presence of ConA (5 μg/ml). Cells were cocultured for 3 days, and pulsed with 1 μCi ^3^H-thymidine 6 h before harvesting.

## Abbreviations

Treg: regulatory T cells;

FTOC: fetal thymus organ culture;

TSLP: thymus stromal-derived lymphopoietin;

mTEC: medullary thymic epithelial cells.

CMJ: cortical-medullary junctions.

APC: antigen presenting cells.

## Authors' contributions

Qi Jiang carried out the whole studies and drafted the manuscript. Hua Su carried out the immuno-staining. Geoffrey Knudsen participated in the Treg function assay. Whitney Helms participated in the real-time PCR study and performed the statistical analysis. Lishan Su conceived of the study, and participated in its design and coordination and helped to draft the manuscript. All authors read and approved the final manuscript.
